# Quantitative optical property mapping of the periocular region using spatial frequency domain imaging

**DOI:** 10.1117/1.JBO.31.9.096005

**Published:** 2026-09-29

**Authors:** Lilangi S. Ediriwickrema, Miranda B. An, Stephanie D. Kirk, John Abdelmalek, Divya Devineni, Shijun Sung, Katherine Chen, Kaylyn C. Mattick, Fiza A. Qureshi, Claire Malley, Jaylen M. Lee, Danny Xie, Gordon T. Kennedy, Bernard Choi, Anthony J. Durkin

**Affiliations:** a University of California, Beckman Laser Institute and Medical Clinic, Irvine, California, United States; b University of California, Department of Ophthalmology, Gavin Herbert Eye Institute, Irvine, California, United States; c Brunson Center for Translational Vision Research, UC Irvine, California, United States; d University of California, Biostatistics, Epidemiology, and Research Design Unit, Irvine, California, United States; e University of California, Department of Biomedical Engineering, Irvine, California, United States; f University of California, Department of Surgery, Irvine, California, United States

**Keywords:** oculofacial, periocular, orbital, spatial frequency domain imaging, optical properties

## Abstract

**Significance:**

Noninvasive characterization of periocular skin optical properties is critical for understanding tissue alterations associated with thyroid eye disease and other orbital adnexal pathologies. Spatial frequency domain imaging (SFDI) provides quantitative, *in vivo* assessment of tissue absorption and scattering, making it a promising tool for clinical evaluation of the periocular region. However, the complex curvature and height variations of orbital anatomy pose unique challenges that may affect measurement accuracy and reproducibility. This study evaluates the feasibility and repeatability of SFDI for periocular imaging in healthy subjects, establishing a foundation for future investigations of disease-related tissue changes.

**Aim:**

The study aims to establish normative SFDI-derived optical properties of periocular skin in the absence of cutaneous disease.

**Approach:**

Twenty-five healthy participants were imaged using the Reflect RS™ SFDI system. Reduced scattering ($\mu_{s}^{\prime }$) and absorption ($\mu_{a}$) coefficients were quantified for predefined regions of interest (ROI): inferior temporal, inferior nasal, and forehead (control). A subset of seven subjects underwent imaging in both frontal and lateral orientations to evaluate the effect of acquisition angle.

**Results:**

Normative ranges for $\mu_{s}^{\prime }$ and $\mu_{a}$ across periocular and forehead ROIs are reported. Imaging angle did not significantly affect optical property estimates. Across the cohort, $\mu_{a}$ increased and $\mu_{s}^{\prime }$ decreased with higher Fitzpatrick scores, consistent with prior literature. We observed that laterality and ROI appear to be significant when measuring optical properties. In addition, $\mu_{s}^{\prime }$ suggests a potential stepwise decline with advancing age.

**Conclusions:**

This study provides the first quantitative characterization of normal periocular skin using SFDI across diverse skin types. Measurements were robust to acquisition angle but varied with pigmentation, underscoring the need to account for Fitzpatrick scores in clinical applications. Establishing normative data is essential for future studies involving periocular disease.

## 1 Introduction

Noninvasive diffuse optical spectroscopy (DOS) encompasses techniques that quantify tissue physiology by analyzing scattering and absorption in the visible and near-infrared spectrum. Spatial frequency domain approaches modulate the light source at various spatial frequencies and analyze the phase shift of the reflected light. Specifically, spatial frequency domain imaging (SFDI) uses previously published light transport models to determine wavelength-dependent tissue reduced scattering ($\mu_{s}^{\prime }$) and absorption coefficients ($\mu_{a}$), which in turn can inform us of subsurface structural and functional tissue properties. SFDI allows for interrogation of tissue volumes up to 5 mm in depth and has demonstrated utility in evaluating burn severity, tissue flap viability following surgical reconstruction, laser treatment efficacy in subjects with port-wine stains, and changes in cerebral blood flow following cardiac arrest.^1^^–^^5^ Seminal work by our group identified changes in spatially resolved maps of tissue hemoglobin and oxygenation in a rat dorsal skin flap following controlled ischemia, reperfusion, and healing.^6^ These advances demonstrate that the measurements collected by SFDI correlate with subsurface tissue regions. Hence, using SFDI, we can evaluate subjects to quantify subsurface vascular changes before they are clinically obvious.

Although prior work has focused on imaging relatively flat anatomical sites such as the inner forearm, SFDI has not been applied to the periocular region, a uniquely contoured, nonplanar anatomical area. The orbit is a bony pyramid composed of four walls: the floor, medial wall, lateral wall, and roof. The frontal, sphenoid, zygomatic, maxillary, palatine, ethmoid, and lacrimal bones make up the bony structures of each orbit, which is separated by paired ethmoid sinuses and the nasal cavity. The base of each orbit measures approximately 4 cm by 3.5 cm. Each orbit is laterally rotated, with parallel medial walls and an orbital apex situated approximately 4.5 to 5 cm posteriorly.^7^ Overlying the orbit are the soft tissue structures of the eyelids, an approximately 1 mm thick structure comprising several layers including epidermis, dermis, orbicularis oculi muscle, levator palpebrae, Mueller muscle, orbital septum, fat compartments, tarsus, and conjunctiva.^8^ The orientation of the eyelids over the bulbar conjunctiva of the globe, as it sits within the orbit, imbues the periocular region with a unique nonplanar geometry (Fig. 1).

**Fig. 1:**
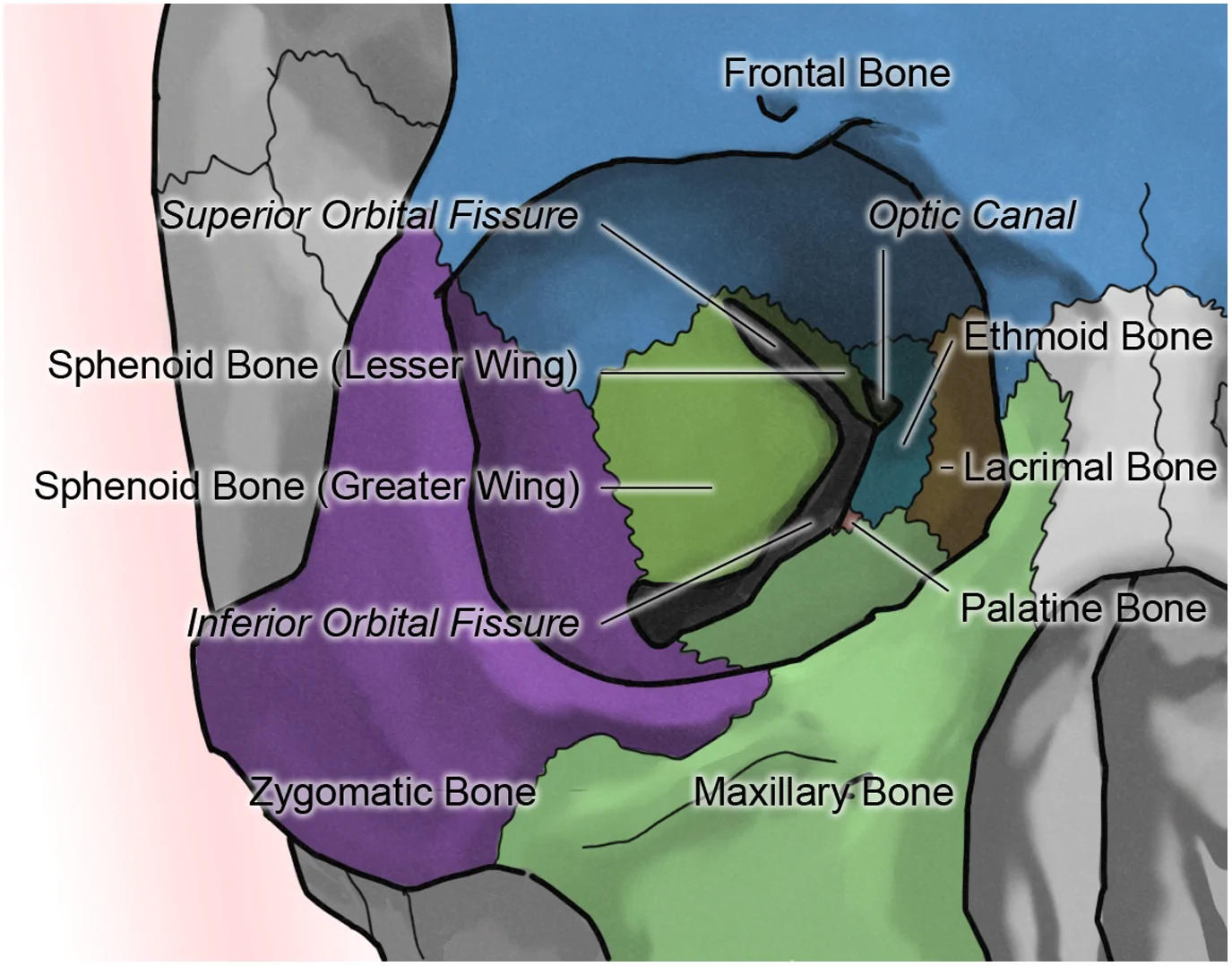
Orbital anatomy highlighting bony landmarks and emphasizing the nonplanar foundation of the periocular and ocular adnexal soft tissue region. Image courtesy of Michael Han, MD.

To date, wide-field quantitative imaging of the periocular region has not been extensively studied. Spatial frequency domain imaging (SFDI) is a promising technology that could address this gap.^1^^–^^5^^,^^9^^–^^12^ However, SFDI measurements along the periocular region may be impacted by height variations and curvature inherent to the periocular region. As an initial step, we recently reported on the use of SFDI to image the periocular region and forehead of a realistic facial phantom.^13^ This study assessed the impact of region of interest location and curvature on optical property measurements, identifying areas with minimal error [e.g., inferior temporal quadrant (ITQ), inferior nasal quadrant (INQ), and forehead] and those with notable measurement variability due to concavity and topographic variation (rostral lateral nasal bridge and central eyelid margin). These findings provide objective knowledge on the capabilities and limitations of SFDI for quantitative periorbital region measurements, paving the way for future clinical applications.

Before applying SFDI to periocular disease states, it is critical to establish normative optical property values and validate region selection in control subjects. Here, we report the first study of *in vivo* periocular optical properties using SFDI in 25 healthy subjects across a range of Fitzpatrick skin types (I-VI), from three key anatomical regions along the periocular and facial region. We characterize $\mu_{a}$ and $\mu_{s}^{\prime }$ along the ITQ, INQ, and FH regions in both “en face” and “side profile” orientations (Fig. 2). We also assess intra-subject reproducibility, as well as the influence of Fitzpatrick score and age. This foundational data set supports future work in periocular disease diagnosis and guidance of therapeutic protocols.

**Fig. 2:**
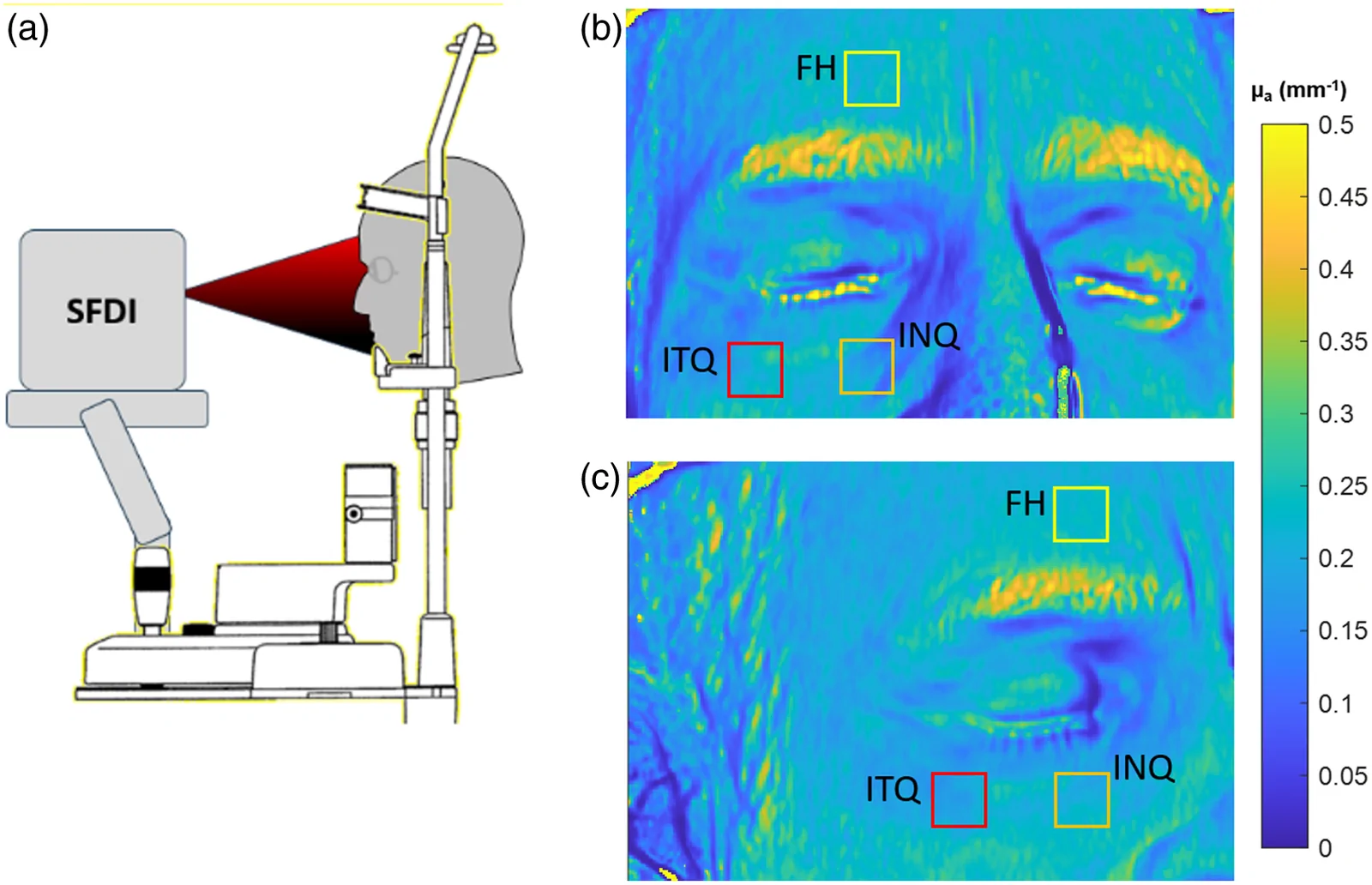
(a) SFDI system consisting of a light source projecting spatially modulated light onto a face positioned in a chin rest, with a resultant (b) “en face” absorption coefficient map and (c) “side profile” absorption coefficient map.

## 2 Materials and Methods

### 2.1 Subject Selection

The experiments were conducted with the full consent of each subject using a protocol approved by the Institutional Review Board (IRB) for human subject measurements at the University of California, Irvine (UCI) (protocol HS#2019-2954). Here, we focused on measuring *in vivo* cutaneous optical properties using SFDI. Twenty-five healthy nonpregnant individuals (ages 19 to 72) were imaged along their periocular region. Subject age, gender, skin color (subjective Fitzpatrick measurement and objective bedside colorimeter score), smoking status, prior medical health, and prior ocular health were reviewed and documented. The Fitzpatrick scale is a clinical grading system comprising six categories primarily based on how an individual’s skin responds to sun exposure. Data collection and evaluation of protected human subject health information throughout the course of this study were HIPAA compliant and adhered to the guidelines set forth by the Declaration of Helsinki.

### 2.2 Subject Imaging

The Reflect RS™ (Modulim, Inc., Irvine, California, United States) was used to perform SFDI by measuring calibrated diffuse reflectance images over a $15\times 20\;{\mathrm{cm}}^{2}$ field of view, with a pixel resolution of ∼0.5  mm. The SFDI light sources consist of eight light emitting diodes (LEDs) with center wavelengths ranging from 470 to 851 nm. For each wavelength, sinusoidal light patterns at five evenly spaced spatial frequencies (from 0 to 0.2/mm) were projected onto the tissue. At each imaging session, a polydimethylsiloxane-based tissue-simulating phantom with known optical properties was imaged under identical lighting conditions for calibration.

In the Advanced Technologies Suite at the Beckman Laser Institute, subjects were instructed to place their head on a custom-made cart-based chin rest with dual forehead apposition to minimize motion artifact (Fig. 2). Corneal protectors were placed over the eyes, and the imaging field of view was manually centered on the periocular region. Each full acquisition (all eight wavelengths and five spatial frequencies) took approximately 30 s, with data processing for each subject completed in under 10 min using accompanying software (Modulim Inc., Irvine, California, United States). Each anatomical region was imaged three times consecutively to mitigate motion artifacts.

### 2.3 SFDI Data Extraction and Analyses

Raw reflectance images were processed using MI-Analyze (Modulim, Inc., Irvine, California, United States) and calibrated against reference phantom images. A well-established Monte Carlo light transport forward model was used to obtain the tissue reduced scattering coefficient ($\mu_{s}^{\prime }$) and absorption coefficient ($\mu_{a}$) at each of the eight wavelengths.^14^ The Monte Carlo model generated a 768 ×768 element look-up-table with coefficient ranges of $0\leq \mu_{a}\leq 3.0\;{\mathrm{mm}}^{-1}$ and $0.01\leq \mu_{s}^{\prime }\leq 4.0\;{\mathrm{mm}}^{-1}$, with anisotropy and refractive index values fixed at 0.8 and 1.4, respectively.^15^ Regions of interest (ROI) were demarcated by a single individual to minimize bias and variability.^13^ These ROI include the inferior nasal (INQ) and inferior temporal quadrants (ITQ) (Fig. 2(b)), as well as the forehead (FH, control). To ensure reproducibility, the ITQ has been defined as the area approximately 1 cm below the lateral canthus, the INQ as the area 1 cm below and temporal to the medial canthus, and the FH as the area 1 cm above the brow on the same side as the imaged periocular region. In addition, intra-subject repeatability and inter-rater reliability were assessed by selecting five subjects randomly, for whom each of the three consecutive acquisitions was analyzed for each ROI by a second rater. The calibration phantom was also imaged and analyzed at three time points over several months during the subject enrollment period.

### 2.4 Statistical Analysis

The optical properties $\mu_{a}$ and $\mu_{s}^{\prime }$ were computed for each ROI to generate eight measured values (four pairs of absorption and scattering values per subject side). SFDI values were summarized using descriptive statistics stratified by age and Fitzpatrick score. We investigated if there were differences in mean optical properties between ROI using linear mixed effects model with ROI and laterality as fixed effects. We then quantified the effect of age on average $\mu_{a}$ and $\mu_{s}^{\prime }$ values with a linear mixed-effects model of the control subjects stratified by age at 851 nm for the ITQ and FH regions. We present uncorrected p-values as a useful summary statistic and will consider a result statistically significant if the p-value does not exceed 0.001. This is to strike a reasonable balance between a family-wise false positive error rate and power due to our sample size.

## 3 Results

### 3.1 Subject Demographics

We imaged 25 human control subjects from 12/2021 until 8/2024. The subjects ranged in age from 19 to 72 years of age and Fitzpatrick 1 to 6. On review of the subject demographic data, a few key trends were identified. Younger subjects predominantly comprised the Fitzpatrick 5 (19 to 25 years) and 6 (24 to 33 years) groups in our control study group. The majority of subjects were Fitzpatrick 2 to 4 (76%) or less than 40 years of age (60%). (Tables 1 and 2).

**Table 1:** Subject demographics by Fitzpatrick score. Twenty-five control subjects without periocular disease were imaged. The majority of subjects comprised Fitzpatrick scores 2 to 4.

Fitzpatrick score	Number of patients (n)	Age range (years)
1	2	34 to 55
2	6	33 to 72
3	8	25 to 68
4	5	23 to 57
5	2	19 to 25
6	2	24 to 33

**Table 2:** Subject demographics by age. Normative periocular imaging data from subjects without cutaneous disease were conducted on a sample size that was predominantly younger, e.g., less than 40 years of age.

Age (years)	Number of patients (n)	Fitzpatrick range
19 to 29	10	3 to 6
30 to 39	5	1 to 6
40 to 49	2	2
50 to 59	4	1 to 4
60 to 69	3	2 to 3
70 to 79	1	1 to 2

### 3.2 Preliminary Repository of Human Control Data: ROI Characterization

The spread of data for each region of interest is presented for 25 control subjects (see Supplementary Tables 1 and 2). At 851 nm, the mean $\mu_{a}$ was 0.02, 0.02, and $0.03\;{\mathrm{mm}}^{-1}$ for the ITQ, INQ, and forehead regions, respectively; the mean $\mu_{s}^{\prime }$ was 1.21, 1.36, and $1.44\;{\mathrm{mm}}^{-1}$ for the ITQ, INQ, and FH ROI, respectively. When further sub-divided by laterality, the mean left $\mu_{a}$ was 0.02, 0.02, and $0.02\;{\mathrm{mm}}^{-1}$ for the ITQ, INQ, and forehead regions, respectively; the mean left $\mu_{s}^{\prime }$ was 1.26, 1.43, and $1.55\;{\mathrm{mm}}^{-1}$ for the ITQ, INQ, and FH ROI, respectively. The mean right $\mu_{a}$ was 0.01, 0.02, and $0.02\;{\mathrm{mm}}^{-1}$ for the ITQ, INQ, and forehead regions, respectively; the mean right $\mu_{s}^{\prime }$ was 1.2, 1.35, and $1.47\;{\mathrm{mm}}^{-1}$ for the ITQ, INQ, and FH ROI, respectively (Table 3). The optical properties measured at 471 nm are also presented in Table 4. Based on a linear mixed effects model with ROI and laterality as fixed effects, the ROI was found to be statistically significant when measuring both $\mu_{a}$ ($\chi^{2}(3)=130.61$, p<.001) and $\mu_{s}^{\prime }$ ($\chi^{2}(3)=239.16$, p<.001) at 851 nm. Laterality, e.g., left side versus right side, was similarly found to be statistically significant when measuring $\mu_{s}^{\prime }$ ($\chi^{2}(1)=14.55$, p<.001), but not for $\mu_{a}$. At 471 nm, the linear mixed effects model with ROI and laterality as fixed effects revealed statistical significance for ROI when measuring both $\mu_{a}$ ($\chi^{2}(3)=20.046$, p<.001) and $\mu_{s}^{\prime }$ ($\chi^{2}(3)=27.447$, p<.001). Laterality, again, was similarly found to be statistically significant when measuring $\mu_{s}^{\prime }$ ($\chi^{2}(1)=11.23$, p<.001), but not for $\mu_{a}$.

**Table 3:** Periocular regions of interest (ROI) optical characteristics. The mean and standard deviation enclosed within parenthesis of the absorption and reduced scattering coefficients for each measured ROI are presented at 851 nm.

n=25	Laterality	ITQ	INQ	Forehead
	Left	0.02 (0.01)	0.02 (0.01)	0.02 (0.01)
$\mu_{a}$ (${\mathrm{mm}}^{-1}$)	Right	0.01 (0.01)	0.02 (0.01)	0.02 (0.01)
	Left	1.26 (0.16)	1.43 (0.19)	1.55 (0.25)
$\mu_{s}^{\prime }$ (${\mathrm{mm}}^{-1}$)	Right	1.20 (0.17)	1.35 (0.20)	1.47 (0.26)

**Table 4:** Periocular regions of interest (ROI) optical characteristics. The mean and standard deviation enclosed within parenthesis of the absorption and reduced scattering coefficients for each measured ROI is presented at 471 nm.

n=25	Laterality	ITQ	INQ	Forehead
	Left	0.49 (0.22)	0.55 (0.35)	0.67 (0.57)
$\mu_{a}$ (${\mathrm{mm}}^{-1}$)	Right	0.46 (0.21)	0.52 (0.29)	0.58 (0.34)
	Left	2.04 (0.45)	2.20 (0.53)	2.44 (0.78)
$\mu_{s}^{\prime }$ (${\mathrm{mm}}^{-1}$)	Right	1.87 (0.38)	2.04 (0.47)	2.23 (0.70)

Five subjects were randomly selected, and each of the three consecutive acquisitions was analyzed for each ROI by a second rater. Each subject was analyzed as a set of three measurements, and these values were averaged across all five subjects, generating mean values for each optical property. In addition, the calibration phantom was imaged at three time points over several months during the subject enrollment period. We report the mean coefficient of variation (CV) as approximately 0.8 to 6.36% for the human controls and 0.125 to 0.972% for the calibration phantom across the 471 and 851 nm measurements (Supplemental Tables 4 and 5). When adjusted for optical property and wavelength, the CV% is approximately 1.98-3.952 for $\mu_{a}$ at 471 nm, 2.127-6.357 for $\mu_{a}$ at 851 nm, 1.083-3.733 for $\mu_{s}^{\prime }$ at 471 nm, and 0.801-3.398 for $\mu_{s}^{\prime }$ at 851 nm. These values are negligible, supporting precision in measurement and intra-subject reliability. Further, the inter-rater agreement (ICC) for the absorption coefficient was ∼0.99 for ITQ, INQ, and the forehead at 851 nm, and the reduced scattering coefficient was 0.92 to 0.99 for ITQ, INQ, and the forehead at 851 nm. These values are very close to 1, indicating high repeatability in measurement (see Supplementary Table 6).

### 3.3 Device Position and Angle of Incidence Does Not Impact *In Vivo* Measurements

To evaluate the potential impact of technician error (i.e., aligning the light source in front of the subject) and/or subject positioning error (i.e., placing subject’s chin along the chinrest), we imaged seven control subjects from both an “en face” and “side profile” vantage point. These subjects ranged in age from 24 to 55 years of age and encompassed Fitzpatrick scores 1, 2, 3, and 6. The findings demonstrate that there is not enough evidence to suggest there is a difference in $\mu_{a}$ and $\mu_{s}^{\prime }$ based on positioning, suggesting minimal impact from subject or camera positioning when using a chinrest (Table 5). Positioning-based analyses suggested differences in the $\mu_{s}^{\prime }$ of the right ITQ, left INQ, right INQ, and left forehead; however, these findings did not withstand the p<0.001 threshold as previously established. See Supplementary Table 3).

**Table 5:** Mean and 99% confidence interval, enclosed within parentheses, for the “en face” and “side profile” capture of optical property measurements across various ROI demonstrate no appreciable differences with p-values >0.05.

ROI	Property	En face	Side profile	Difference	p-value
ITQ	$\mu_{a}$	0.01 (0.001,0.02)	0.01 (0.002,0.02)	0 (−0.003,0.004)	0.62
ITQ	$\mu_{s}^{\prime }$	1.24 (0.97,1.51)	1.26 (0.96,1.56)	−0.02 (−0.13,0.09)	0.37
INQ	$\mu_{a}$	0.01 (0.01,0.02)	0.01 (0.003,0.02)	0.001 (−0.004,0.005)	0.44
INQ	$\mu_{s}^{\prime }$	1.41 (0.90,1.91)	1.38 (0.76,2.00)	0.03 (−0.19,0.25)	0.44
Forehead	$\mu_{a}$	0.01 (0.003,0.02)	0.01 (0.004,0.02)	−0.001 (−0.006,0.005)	0.49
Forehead	$\mu_{s}^{\prime }$	1.52 (1.07,1.98)	1.44 (1.07,1.81)	0.08 (−0.14,0.31)	0.07

### 3.4 Potential Challenges in Image Acquisition due to Cutaneous Melanin Concentration:

Fitzpatrick score, itself, provides somewhat of a challenge in extracting accurate and precise optical property measurements, as previously described.^15^ Our control subjects similarly exhibit increases in the magnitude of the wavelength-dependent $\mu_{a}$ coefficients with increasing Fitzpatrick score, whereas the value of the wavelength-dependent $\mu_{s}^{\prime }$ decreases with increasing Fitzpatrick score^15^ (Fig. 3). The $\mu_{a}$ and $\mu_{s}^{\prime }$ were similar over the longer wavelength spectra (∼691 to 851 nm), suggesting that longer wavelengths may overcome the challenges associated with cutaneous melanin concentration, as also previously described in the literature^16^ (Figs. 3 and 4, Table 6, Supplementary Tables 1 and 2).

**Fig. 3:**
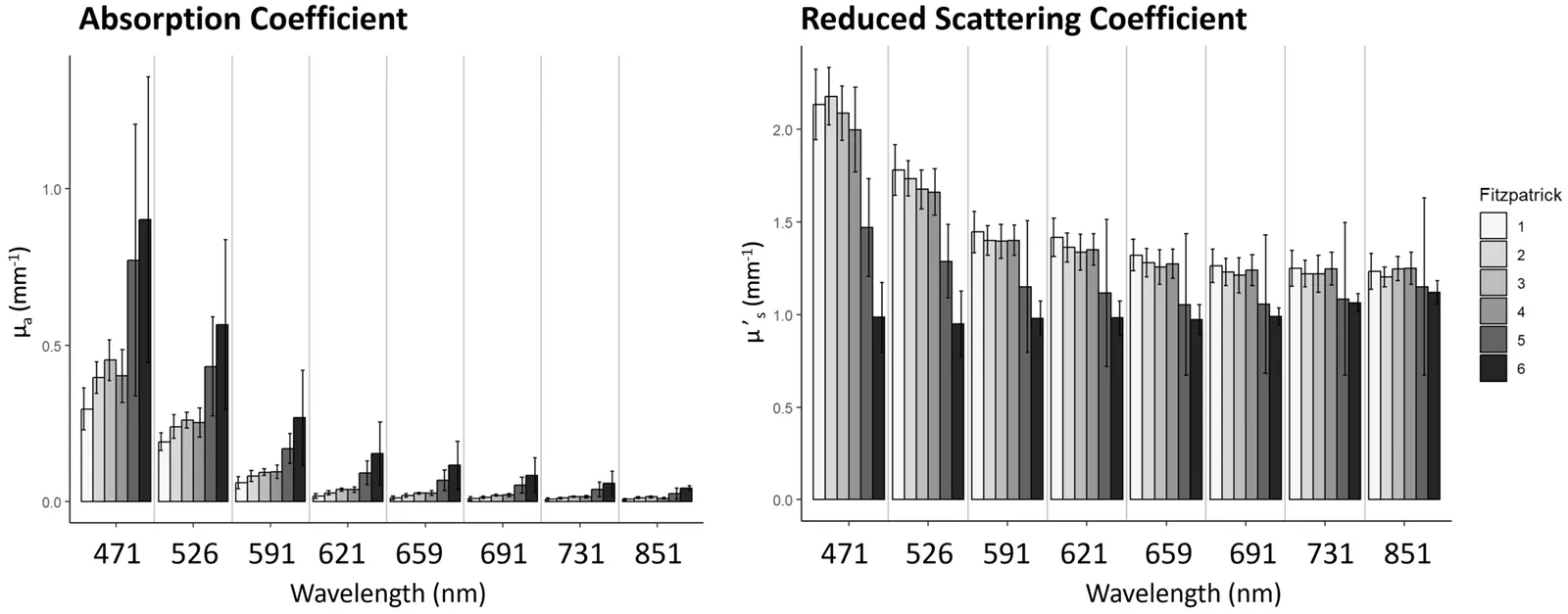
Box and whisker plots of $\mu_{a}$ and $\mu_{s}^{\prime }$ for ITQ, stratified by Fitzpatrick, at all center wavelengths 471 to 851 nm. $\mu_{a}$ increases with increasing Fitzpatrick score; however, the variance is minimized at longer wavelengths. The $\mu_{s}^{\prime }$ decreases with increasing Fitzpatrick score; similarly, the variance is minimized at longer wavelengths.

**Fig. 4:**
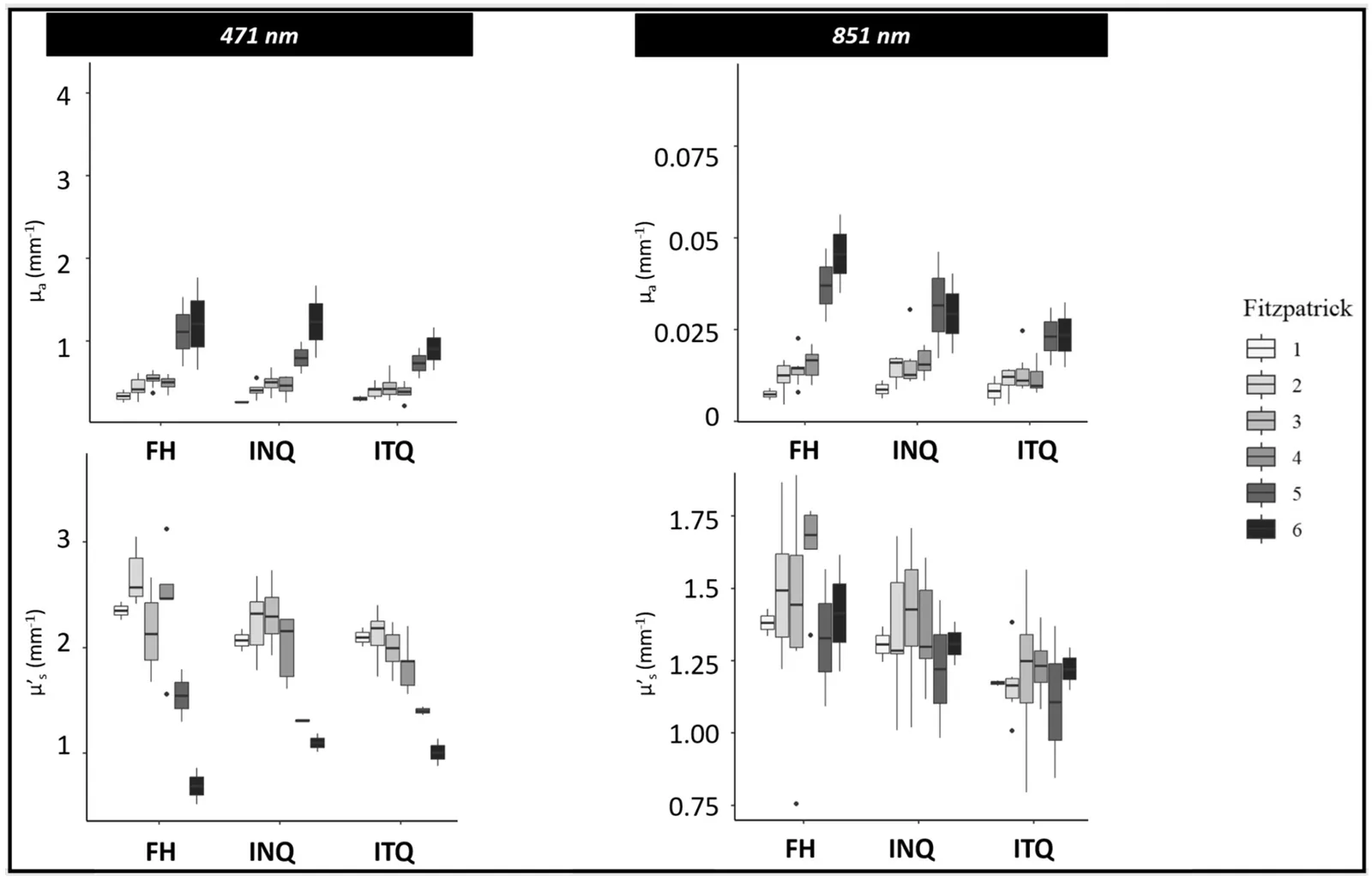
Box and whisker plots of $\mu_{a}$ and $\mu_{s}^{\prime }$ for each right-sided periocular ROI, stratified by Fitzpatrick, at 851 and 471 nm. The observed increase in $\mu_{a}$ and decrease in $\mu_{s}^{\prime }$ with increasing Fitzpatrick scores is more significant in amplitude at shorter wavelengths (e.g., 471 nm) as compared to longer wavelengths (e.g., 851 nm).

**Table 6:** Median $\mu_{a}$ and $\mu_{s}^{\prime }$ with the first and third interquartiles enclosed within parentheses for ITQ for wavelengths 471 and 851 nm reveal similar values for subjects at 851 nm.

Fitzpatrick	$\mu_{s}^{\prime }$-471 nm	$\mu_{s}^{\prime }$-851 nm	$\mu_{a}$-471 nm	$\mu_{a}$-851 nm
1	2.07 (2.03 to 2.23)	1.23 (1.17 to 1.23)	0.32 (0.29 to 0.36)	0.008 (0.007 to 0.009)
2	2.23 (2.12 to 2.34)	1.18 (1.12 to 1.28)	0.27 (0.27 to 0.30)	0.009 (0.007 to 0.011)
3	2.14 (1.91 to 2.5)	1.26 (1.10 to 1.33)	0.38 (0.33 to 0.50)	0.013 (0.012 to 0.016)
4	1.94 (1.79 to 2.20)	1.26 (1.19 to 1.37)	0.41 (0.30 to 0.42)	0.014 (0.011 to 0.015)
5	1.40 (1.37 to 1.51)	1.16 (0.92 to 1.39)	0.48 (0.42 to 0.55)	0.014 (0.012 to 0.020)
6	1.28 (1.09 to 1.45)	1.10 (1.02 to 1.22)	0.45 (0.36 to 0.58)	0.013 (0.010 to 0.016)

### 3.5 A Role for Aging in Affecting Tissue Measurement:

Controls were further sub-stratified by age, and our data suggest that $\mu_{s}^{\prime }$ may potentially decrease with increasing age at 851 nm (Fig. 5), as previously described.^17^ The age groups were collapsed to maintain adequate approximate per-cell sample size when analyzing across various Fitzpatrick scores. The longer 851 nm wavelength was chosen given its relative resilience to the impact of melanin on $\mu_{s}^{\prime }$ and $\mu_{a}$. Age effects were re-estimated using linear mixed-effects models with CR2 cluster-robust standard errors, clustering on subject ID, to account for repeated bilateral measurements and heteroscedasticity in the residuals (Satterthwaite-adjusted degrees of freedom; all confidence intervals reported at the 99% level). At 851 nm, the age-adjusted model for the ITQ region revealed a negative age slope of $-0.0042\;{\mathrm{mm}}^{-1}$ per year (robust p=0.056; 99% CI: −0.010, 0.002; marginal $R^{2}=0.23$) (Fig. 6), suggesting that $\mu_{s}^{\prime }$ decreases by approximately $0.0042\;{\mathrm{mm}}^{-1}$ with every additional year of age. A similar negative trend was observed for the forehead region at 851 nm (slope: $–0.0062\;{\mathrm{mm}}^{-1}/\mathrm{year}$; robust p=0.129; 99% CI: −0.018, 0.005; marginal $R^{2}=0.16$).

**Fig. 5:**
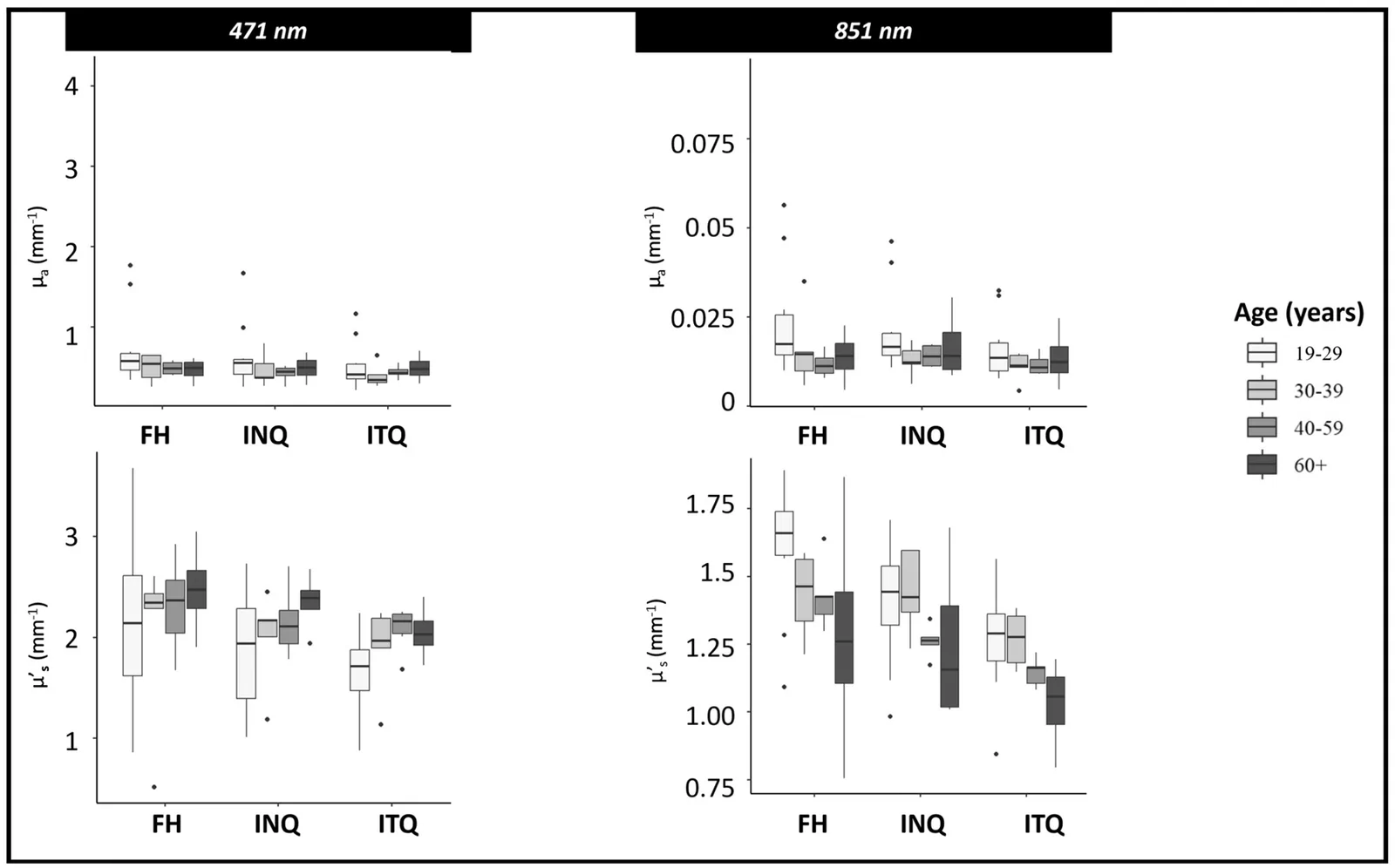
Box and whisker plots of $\mu_{a}$ and $\mu_{s}^{\prime }$ for each right-sided periocular ROI, stratified by age, reveal a decrease in $\mu_{s}^{\prime }$ trend with increasing age for ROI ITQ, INQ, and FH at 851 nm.

**Fig. 6:**
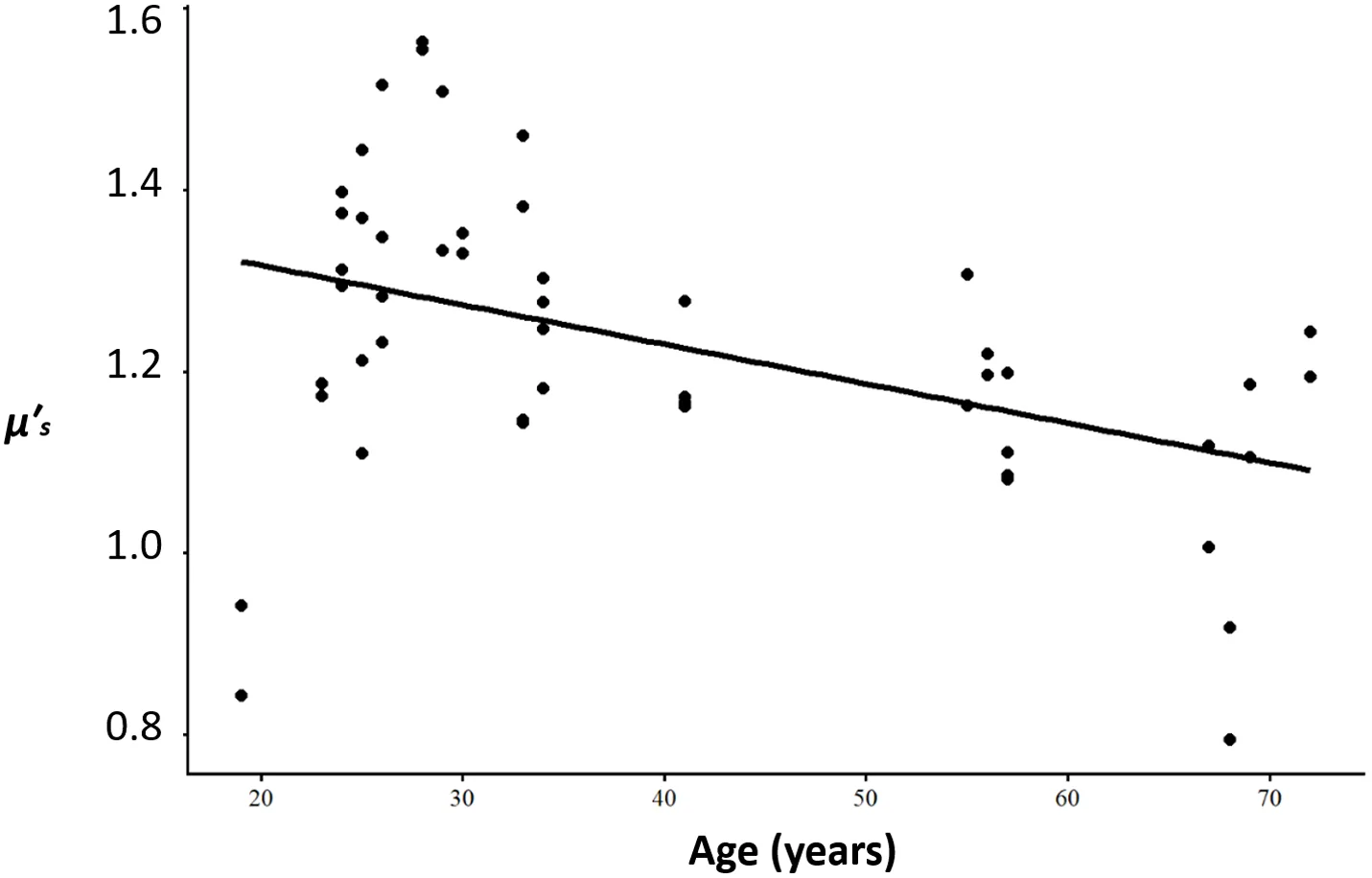
A linear mixed-effects model of the control subjects stratified by age at 851 nm for the ITQ region revealed a negative slope (−0.0042) signifying μ’s decreases by $0.0042\;{\mathrm{mm}}^{-1}$ with each year of age (robust p=0.056, 99% CI: –0.010, 0.002; marginal $R^{2}=0.23$).

In contrast, at 471 nm, a positive age slope of $0.0097\;{\mathrm{mm}}^{-1}$ per year was observed for the ITQ region (robust p=0.074; 99% CI: −0.006, 0.025; marginal $R^{2}=0.18$), with a corresponding positive trend at the forehead (slope: $+0.0129\;{\mathrm{mm}}^{-1}/\text{year}$; robust p=0.150; 99% CI: −0.013, 0.039; marginal $R^{2}=0.07$), consistent with an increase in $\mu_{s}^{\prime }$ with advancing age at this shorter wavelength. Although the opposing directional trends across wavelengths are consistent with prior observations, none of these estimates achieves conventional statistical significance (p<0.05) under the more conservative robust inference framework, and all 99% confidence intervals include zero. As such, none meets the predefined significance threshold of p<0.001, and these findings should be regarded as exploratory trends only. However, it remains unclear whether there is truly an impact due to age or if the data are biased by having a younger cohort of subjects with higher represented Fitzpatrick scores.

### 3.6 Potential Multivariate Role of Age and Fitzpatrick

The ITQ periocular region was again further evaluated by dividing each age cohort into Fitzpatrick scores. Within each age cohort, a similar trend of increasing $\mu_{a}$ alongside increasing Fitzpatrick score was observed. Similarly, a decrease in $\mu_{s}^{\prime }$ was observed with increasing Fitzpatrick score within each age cohort. The trend of decreasing $\mu_{s}^{\prime }$ with increasing age was also observed as a general trend (Fig. 7). The age groups were collapsed to maintain an adequate approximate per-cell sample size when analyzing across various Fitzpatrick scores.

**Fig. 7:**
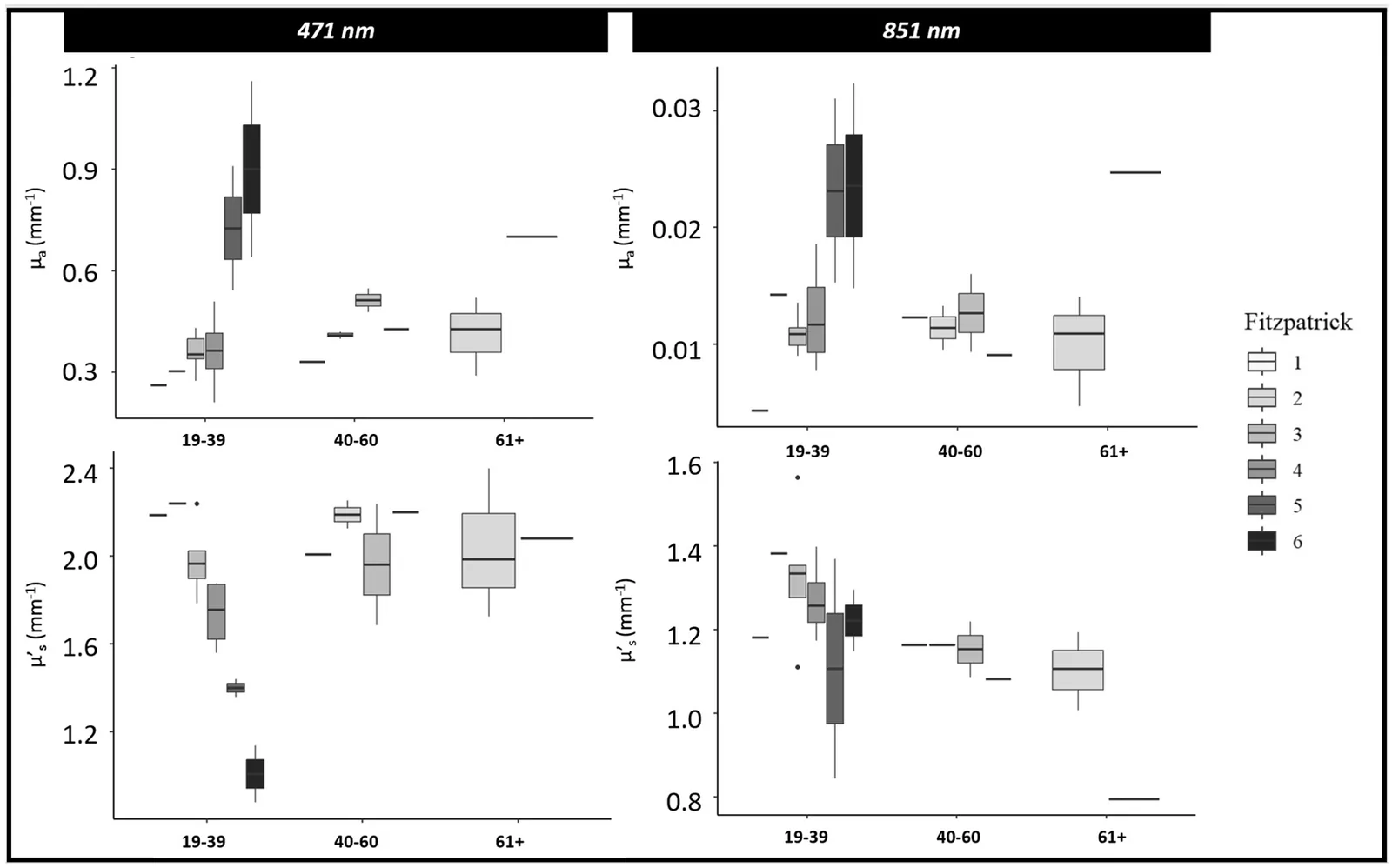
Box and whisker plots of $\mu_{a}$ and $\mu_{s}^{\prime }$ for right-sided ITQ, stratified by Fitzpatrick and age, at 471 and 851 nm. A similar trend of decreasing $\mu_{s}^{\prime }$ with increasing Fitzpatrick and increasing age was observed at 851 nm.

## 4 Discussion

Spatial frequency domain imaging is poised to interrogate and quantify wavelength-specific $\mu_{a}$ and $\mu_{s}^{\prime }$ towards characterizing normal variants of tissue physiology. In this study, SFDI was able to characterize normal ranges of $\mu_{a}$ and $\mu_{s}^{\prime }$ for various age groups over all Fitzpatrick scores along the periocular region, as compared with the forehead region. The periocular region of interest, upon being subdivided into two quadrants: ITQ and INQ, in addition to the FH control, was found to be statistically significant based on a linear mixed effects model when measuring $\mu_{a}$ and $\mu_{s}^{\prime }$. This suggests that a ROI is associated with different optical properties, suggesting variable measured optical properties between each periocular/facial region. 

Significant laterality differences were observed in $\mu_{s}^{\prime }$ at 471 and 851 nm, with the left (more sun-exposed) side consistently exhibiting higher $\mu_{s}^{\prime }$ than the right side. In contrast, $\mu_{a}$ did not differ significantly between sides at either 471 or 851 nm. This wavelength-dependent pattern is consistent with differential sensitivity to photoaging-related changes, where 471 nm reflects both epidermal melanin content and superficial structural alterations, and 851 nm primarily reflects deeper dermal microstructural remodeling independent of pigmentation. 

Beyond cumulative sun exposure, additional factors may contribute to the observed asymmetries. These include potential systematic acquisition biases, such as slight differences in subject positioning, camera geometry, or illumination angle during imaging, as well as behavioral asymmetries (e.g., dominant-hand positioning or habitual head orientation). Nevertheless, the persistence of laterality differences in $\mu_{s}^{\prime }$ at 851 nm—where melanin-related confounding is minimal—supports the interpretation that SFDI may be sensitive to subtle dermal microstructural changes, potentially reflecting early collagen and extracellular matrix remodeling associated with chronic environmental exposure. 

The dual forehead and chin rest approximation system, furthermore, allows for reproducible positioning of the subject in front of the camera system. Minimal changes in positioning (such as “en face” versus “side profile” image capture) do not appear to impact SFDI measurement accuracy along the ITQ, INQ, and FH, suggesting that the geometry of the selected ROIs along the periocular region has acceptably little influence on SFDI measurements, as previously observed in studies evaluating a solid tissue-simulating facial phantom.^13^ Further, it appears imaging angle does not significantly affect optical property estimates. 

An increase in $\mu_{a}$ and decrease in $\mu_{s}^{\prime }$ for subjects with higher Fitzpatrick score was observed, irrespective of age, as previously described by Phan et al., Saager et al., and Jonasson et al.^15^^,^^18^^,^^19^ It is postulated that the decrease in $\mu_{s}^{\prime }$ is attributed to the higher melanin concentration at the higher Fitzpatrick scores, in turn resulting in decreased probing depth due to earlier, or increased, absorption by melanin. In contrast, the lower Fitzpatrick scores allowed deeper penetration of photons to the dermal matrix, which amplify the $\mu_{s}^{\prime }$ particularly in the shorter, visible wavelengths. Phan and colleagues also suggest the depth of penetration is inherently deeper along the near-infrared wavelength range, bypassing the impact of variable melanin concentration as found in the epidermis.^15^ Taken together, these inaccuracies were less significant at longer wavelengths, where melanin plays less of a role. 

Age, itself, may influence measurement as a decrease in $\mu_{s}^{\prime }$ is observed with advancing age. The findings, however, are exploratory and do not meet statistical significance, highlighting an area for more rigorous study through increasing the sample size. This is likely due to loss of elastin and collagen, in addition to changes of the underlying fat pads and bony skeleton, particularly along the periocular region. Not only is there bony resorption, but there is a lateral inferior shift of the lateral and inferior orbital rim, leading to a decrease in the maxillary angle and an increase in the pyriform angle.^20^ There is also inferior migration and deflation of the facial fat compartments, and a loss of the smooth transition between the upper eyelid and brow, as well as the lower eyelid and cheek.^21^^,^^22^ Further, facial retaining ligaments such as the orbitomalar, zygomatico-cutaneous, and upper masseteric ligaments begin to descend as the skin and subcutaneous tissue lose elasticity. This in and of itself can lead to the development of a tear trough deformity and malar bags.^23^ The differences observed at 471 nm are likely due to the depth of penetration being more superficial, resulting in a more superficial sampling volume, as compared with 851 nm, where potentially more information regarding tissue structure can be conveyed.^24^

Fitting a multivariable model that simultaneously adjusts for both age and Fitzpatrick score is not feasible in this sample due to substantial collinearity between the two predictors and sparse per-cell counts. In the dataset, participants with higher Fitzpatrick scores (darker skin tones, scores 5 and 6) are predominantly younger (teens to early thirties), while the majority of older participants (age 40 and above) cluster at Fitzpatrick scores 2 through 4. This creates a strong empirical correlation between age and Fitzpatrick score (Pearson r approaching −0.5 to −0.7 depending on the subsample), inflating the variance of both regression coefficients and making it difficult to independently estimate the effect of age after holding Fitzpatrick score constant, and vice versa. In addition, it could be possible that the effect of age varies depending on skin tone, though we lack data to verify this interaction. With only 25 unique subjects and a limited range of Fitzpatrick categories at each age stratum, the model would be severely underpowered, variance inflation factors (VIF) would exceed acceptable thresholds, and confidence intervals on both age and Fitzpatrick effects would be too wide to be clinically meaningful. The two variables are therefore treated separately in descriptive, exploratory, analyses rather than combined in a single adjusted model. The opposing age-related regression trends in $\mu_{s}^{\prime }$ at 471 and 851 nm are consistent with the wavelength-dependent sensitivity of $\mu_{s}^{\prime }$ to different scatterer-size distributions in aging skin. Scattering at 851 nm is more strongly influenced by larger dermal structures and forward-directed Mie-regime scattering; age-related loss of organized collagen bundles and reduced dermal packing density would therefore be expected to decrease $\mu_{s}^{\prime }$ at this wavelength, a trend consistent with prior reports of declining NIR $\mu_{s}^{\prime }$ with age in skin.^25^ In contrast, scattering at 471 nm is more sensitive to smaller-scale refractive-index fluctuations and Rayleigh-like contributions from submicron scatterers. Age-related microstructural remodeling, including increased collagen cross-linking, accumulation of lipofuscin granules, and reorganization of dermal microdomains, would be expected to increase the number density and refractive-index contrast of these smaller scatterers, raising $\mu_{s}^{\prime }$ at shorter wavelengths.^26^^–^^29^ A wavelength-dependent sign reversal in $\mu_{s}^{\prime }$ is therefore the expected signature of an aging-related shift in the balance of scatterer size distribution and refractive-index contrast. We emphasize, however, that both the 471 and 851 nm regressions in our cohort do not meet our prespecified significance threshold and should be regarded as a hypothesis to be validated in larger, age-stratified studies. 

Limitations of our study include as stated that most of the younger age cohort comprised most of the higher Fitzpatrick scores, which can skew the optical property results. Specifically, the younger subjects who included higher Fitzpatrick scores demonstrated a larger spread of $\mu_{a}$ values, which became a tighter fit when Fitzpatrick 5 to 6 controls were excluded. Future testing would require additional inclusion of a more representative population within this and older age groups. Fitzpatrick score, itself is a limitation, as periocular pigmentation may not fully reflect an individual’s overall skin phototype as assessed by the classification scheme. The relatively small sample size also precluded sex-stratified analyses, and potential sex-related differences in skin characteristics and SFDI measurements could not be evaluated. Finally, we present uncorrected p-values for the paired comparisons on the SFDI values by region. There is risk of inflated type I error rates for concluding significant differences based solely on these results that should be confirmed in future work.

## 5 Conclusion

As a wide-field imaging modality, SFDI has the ability to penetrate approximately 5 mm in depth, positioning it to capture more tissue data when compared with traditionally used *in vivo* clinical imaging techniques. We present the first normative database of optical property measurements of the periocular region in control subjects. SFDI can now be applied to differentiate tissue afflicted by disease and its response to various treatment modalities when comparing measured optical properties from pathologic tissue as compared with our age-matched and Fitzpatrick-matched controls.

## Supplementary Material

10.1117/1.JBO.31.9.096005.s01

## Data Availability

The datasets generated and/or analyzed during the current study are available upon reasonable request.
