# Supplementary material for: Quantitative optical property mapping of the periocular region using spatial frequency domain imaging
Source: J Biomed Opt. 2026 Sep 29;31(9):096005. doi: 10.1117/1.JBO.31.9.096005 (PMC13621795; doi:10.1117/1.JBO.31.9.096005)
Supplement: Supplementary file 1 [file JBO_031_096005_SD001.docx]

**Supplemental Table 1 (below)**. Median µ_a_ (units of 1/mm) with first and third interquartile enclosed within parentheses at 99% confidence intervals (CI) for ITQ over wavelengths 471-851 nm for patients with Fitzpatrick score 1-6, further sub-divided by laterality, left and right.

**Supplemental Table 2 (below)**. Median µ’_s_ (units of 1/mm) with first and third interquartile enclosed within parentheses at 99% CI for ITQ over wavelengths 471-851 nm for patients with Fitzpatrick score 1-6, further sub-divided by laterality, left and right.

**Supplemental Table** **3 (below)**. Median with first and third interquartile enclosed within parentheses at 99% CI for the “en face” and “side profile” capture of optical property measurements (units of 1/mm) for various ROI. Laterality-based analyses suggested differences in the µ’_s_ of the right ITQ, left INQ, right INQ, and left forehead; however, these findings did not withstand the p < 0.001 threshold as previously established.

**Supplemental Table 4.** The mean CV (%) and median CV (%) are summarized across all retained cases and are presented separately by **laterality** (left, right) for patients.

| Laterality | Parameter (mm^-1^) | Wavelength (nm) | ROI | Mean Coefficient of Variation % | Median Coefficient of Variation |
| --- | --- | --- | --- | --- | --- |
| Left | μ_a_ | 471 | ITQ | 1.98 | 2.048 |
|  |  |  | INQ | 1.986 | 2.323 |
|  |  |  | FOREHEAD | 3.173 | 1.987 |
|  |  | 851 | ITQ | 4.66 | 3.387 |
|  |  |  | INQ | 2.127 | 2.081 |
|  |  |  | FOREHEAD | 3.498 | 2.553 |
|  | μ’_s_ | 471 | ITQ | 1.904 | 1.742 |
|  |  |  | INQ | 2.84 | 2.131 |
|  |  |  | FOREHEAD | 3.733 | 4.006 |
|  |  | 851 | ITQ | 1.388 | 0.777 |
|  |  |  | INQ | 1.322 | 1.403 |
|  |  |  | FOREHEAD | 0.801 | 0.521 |
| Right | μ_a_ | 471 | ITQ | 2.45 | 2.154 |
|  |  |  | INQ | 2.508 | 2.754 |
|  |  |  | FOREHEAD | 3.952 | 1.114 |
|  |  | 851 | ITQ | 4.508 | 4.24 |
|  |  |  | INQ | 3.581 | 4.14 |
|  |  |  | FOREHEAD | 6.357 | 3.557 |
|  | μ’_s_ | 471 | ITQ | 1.083 | 1.118 |
|  |  |  | INQ | 2.867 | 1.657 |
|  |  |  | FOREHEAD | 3.15 | 2.467 |
|  |  | 851 | ITQ | 1.098 | 0.78 |
|  |  |  | INQ | 3.398 | 1.563 |
|  |  |  | FOREHEAD | 1.577 | 0.65 |

**Supplemental Table 5.** The mean CV (%) and median CV (%) are summarized for the calibration phantom imaged at three distinct time-points over several months during the patient enrolment period.

| Parameter (mm^-1^) | Wavelength (nm) | ROI | Mean Coefficient of Variation % | Median Coefficient of Variation |
| --- | --- | --- | --- | --- |
| μ_a_ | 471 | ITQ | 0.972 | 0.805 |
|  |  | INQ | 0.853 | 0.72 |
|  |  | FOREHEAD | 0.393 | 0.406 |
|  | 851 | ITQ | 0.352 | 0.248 |
|  |  | INQ | 0.459 | 0.614 |
|  |  | FOREHEAD | 0.386 | 0.326 |
| μ’_s_ | 471 | ITQ | 0.24 | 0.269 |
|  |  | INQ | 0.303 | 0.315 |
|  |  | FOREHEAD | 0.193 | 0.201 |
|  | 851 | ITQ | 0.134 | 0.145 |
|  |  | INQ | 0.246 | 0.262 |
|  |  | FOREHEAD | 0.125 | 0.155 |

**Supplemental Table 6.** The inter-rater agreement (ICC) with a 95% Confidence Interval comparing ROIs selected for five patients by two raters.

| **Optical Property (mm^-1^)** | **Laterality** | **ROI** | **ICC (95% CI)** |
| --- | --- | --- | --- |
| **μ_a_** | Left | ITQ | 0.993 (0.933, 0.999) |
|  |  | INQ | 0.994 (0.940, 0.999) |
|  |  | Forehead | 0.998 (0.977, 1.000) |
|  | Right | ITQ | 0.998 (0.984, 1.000) |
|  |  | INQ | 0.986 (0.866, 0.999) |
|  |  | Forehead | 0.994 (0.945, 0.999) |
| **μ’_s_** | Left | ITQ | 0.942 (0.447, 0.994) |
|  |  | INQ | 0.988 (0.884, 0.999) |
|  |  | Forehead | 0.980 (0.807, 0.998) |
|  | Right | ITQ | 0.944 (0.467, 0.994) |
|  |  | INQ | 0.920 (0.229, 0.992) |
|  |  | Forehead | 0.987 (0.875, 0.999) |
